# Heritability and responses to high fat diet of plasma lipidomics in a twin study

**DOI:** 10.1038/s41598-017-03965-6

**Published:** 2017-06-16

**Authors:** Turid Frahnow, Martin A. Osterhoff, Silke Hornemann, Michael Kruse, Michal A. Surma, Christian Klose, Kai Simons, Andreas F. H. Pfeiffer

**Affiliations:** 1 Department of Clinical Nutrition, German Institute of Human Nutrition Potsdam-Rehbruecke, Nuthetal, Germany; 2 German Center for Diabetes Research (DZD), Nuthetal, Germany; 3 Department of Endocrinology, Diabetes and Nutrition, Charité – University of Medicine, Berlin, Germany; 4 Lipotype GmbH, Dresden, Germany

## Abstract

Lipidomics have a great potential as clinical tool for monitoring metabolic changes in health and disease. Nevertheless hardly anything is known about the heritability of lipids. Therefore, it is necessary to clarify how and how much we can affect these progresses in individuals. In our interventional twin study (46 healthy, non-obese twin pairs) we investigated the lipid profile in plasma samples after switching from a low fat diet to an isocaloric high fat diet (HFD) to characterize the metabolic adaptation. Additionally we used the ACE model for Additive genetics, Common and unique Environment as well as linear mixed modelling to analyse the heritability of lipids. The heritability of lipids varied between 0–62% and applied to lipid species rather than to lipid classes. Phospholipids showed the highest inheritance. In addition, sex, body mass index (BMI) and age were important modifiers. The lipid profile changed already after one week of HFD and diverged further after 5 weeks of additional HFD. Basal concentrations of specific lipids within phospholipids are strongly inherited and are likely to be associated with heritable disease risks. BMI, sex and age were major modifiers. Nutrition strongly alters specific lipid classes, and has to be controlled in clinical association studies.

## Introduction

Not only does the analysis of the lipidome reveal a remarkable diversity of lipids, but its composition can also be linked directly to health and disease status^1^. Previously, researchers associated reduced concentrations of specific ether lipids in the brain with Alzheimer’s disease and Down syndrome^2, 3^. Metabolic disorders such as obesity, metabolic syndrome and diabetes as well as atherosclerosis and cardiovascular diseases were related to changes in the lipid profile of affected patients^1, 4, 5^. Previous approaches were based on determination of major lipids such as cholesterol and triglycerides. The much higher resolution of modern lipidomics technologies allows for more detailed insights into metabolic changes.

Apart from the opportunity to use lipidomics for monitoring diseases and prediction of metabolic disease risks, lipidomics may elucidate the effect and interplay of the different lipids and allow new insights into mechanisms underlying metabolic changes. Important properties of lipids relate to their content of saturated, mono-, and polyunsaturated fatty acids, since their biological effects and physical properties differ^6^ as well as their head groups or backbones.

Several studies show that lysophosholipids (like lysophosphatidic acid) are able to evoke and modulate immune responses^7^. Heimerl *et al*.^8^ revealed also a pro-inflammatory effect of lower lysophosholipid concentrations and Sigruener *et al*.^9^ demonstrated a beneficial role of specific lysophosphatidylcholines.

Nevertheless individual responses to higher fat intake may vary considerably, possibly reflecting nutrigenetic differences between individuals. Several studies already investigated the heritability of classical clinical markers such as total cholesterol, HDL and LDL and found high proportions of heritability^10, 11^, but – in contrast to lipoproteins - hardly anything is known about the heritability of molecular lipid species in humans. Scheitz *et al*.^12^ suggested that the lipid profile of *D*. *melanogaster* is influenced to a large extent by genetic factors, whereas proportions of odd and even numbered fatty acids were more environmentally influenced. Similar results were reported for the San Antonio Family Heart Study for the human plasma lipidome in Mexican American families^13^.

High fat diets enriched in saturated fatty acids are thought to be unhealthy by inducing dyslipidemia and insulin resistance, increasing vascular inflammation and promoting obesity^14–16^. We postulate that individuals may be able to compensate the unfavourable effects of high fat diets (HFD) and that the changes in a comprehensive lipid profile are not only part of this regulation but are also heritable.

We therefore undertook a study in mono- and dizygotic twins to characterize the metabolic adaptation after switching from a low fat to a high fat Western-style diet based on intakes, which are compatible with current food patterns. The study was performed under isocaloric, weight stable conditions in healthy subjects without changes of physical activity to avoid confounding by weight change effects.

## Results

### Subject characteristics

All 46 twin pairs shown in Table 1 completed the study. The physiological characteristics of the cohort with respect to the study are summarized in Table 2. The slight, but significant increase in body weight did not impair the success of the isocaloric study design and seemed to be sex specific. The significant difference between the first two clinical investigation days LF/HF1 (LF – after six weeks low-fat diet; HF1 – after one week high-fat diet) and the clinical investigation day after six weeks of high-fat diet (HF6) averaged 0.5 kg, which is within a range of natural fluctuation. Apart from this neither the waist-to-hip ratio (WHR) nor the unadjusted or age and sex adjusted body composition changed significantly. Therefore the effects of weight gain or loss were reduced to a minimum in our results.

**Table 1:** Screening clinical parameters of the cohort (n = 92).

	median	IQR_0.25,0.75_
Sample size [n]	92	
Male/female [n]	34/58	
Mono-/dizygotic [n]	68/24	
Age [y]	25	[21, 43]
BMI [kg/m^2^]	22.5	[20.9, 24.6]
Waist-to-hip ratio [ ]	0.8	[0.76, 0.86]
Weight [kg]	64.1	[59.3, 76.9]
Pulse [bpm]	67	[62, 73]
Systolic BP [mm Hg]	117	[109, 126]
Diastolic BP [mm Hg]	74	[68, 80]
Fasting insulin [mU/L]	3.88	[2.94, 5.86]
Fasting glucose [mmol/L]	4.31	[4.00, 4.63]
HbA1c [%]	5	[4.8, 5.3]
Total cholesterol [mmol/L]	4.53	[3.84, 5.29]
HDL cholesterol [mmol/L]	1.28	[1.15, 1.60]
LDL cholesterol [mmol/L]	2.68	[2.16, 3.24]
Triglycerides [mmol/L]	0.94	[0.67, 1.20]
CRP [mg/dL]	0.5	[0.18, 1.48]

Values are given as median and (Q_0.25_, Q_0.75_).

**Table 2:** Changes of clinical parameters of the cohort (n = 92) during the study.

	LF		HF1		HF6		P-value
	median	IQR_0.25,0.75_	median	IQR_0.25,0.75_	median	IQR_0.25,0.75_	
BMI [kg/m^2^]	22.2	[20.6, 24.2]^a^	22.1	[20.6, 24.0]^a^	22.5	[20.6, 24.4]^b^	2.71 × 10^−6^
Waist-to-hip ratio [ ]	0.8	[0.75, 0.85]	0.8	[0.75, 0.84]^a^	0.79	[0.74, 0.84]	0.269
weight [kg]	64.0	[58.6, 74.9]^a^	63.4	[59.1, 74.5]^a^	64.2	[58.6, 76.2]^b^	9.26 × 10^−6^
Fasting insulin [mU/L]	3.9	[2.83, 5.55]^a^	4.75	[3.45, 6.63]^b^	4.55	[2.93, 5.86]^a,b^	0.006
Fasting glucose [mmol/L]	5.03	[4.77, 5.56]	5.18	[4.77, 5.53]	5.13	[4.75, 5.58]	0.550
HOMA-IR []	0.90	[0.61, 1.36]^a^	1.09	[0.75, 1.53]^b^	1.08	[0.64, 1.41]^a,b^	0.012
HbA1c [%]^a^	5.0	[4.8, 5.2]	5.1	[4.8, 5.2]	5.1	[4.9, 5.4]	0.040
Total cholesterol [mmol/L]	4.26	[3.63, 4.86]^a^	4.48	[3.88, 4.95]^b^	4.66	[3.95, 5.35]^c^	8.46 × 10^−11^
HDL cholesterol [mmol/L]	1.18	[1.01, 1.48]^a^	1.27	[1.11, 1.56]^b^	1.36	[1.11, 1.64]^c^	1.71 × 10^−13^
LDL cholesterol [mmol/L]	2.45	[2.09, 3.06]^a^	2.70	[2.14, 3.26]^b^	2.78	[2.23, 3.33]^c^	4.71 × 10^−8^
LPS [EU/ml]	0.08	[0.05, 0.11]	0.08	[0.05, 0.10]	0.08	[0.06, 0.11]	0.265

The different clinical investigation days are compared with a one-way, type III, repeated measures analysis of variance with Bonferroni adjustment. Different letters in the cells clarify significant differences between the CIDs after post-hoc test. Values are given as median, (Q_0.25_, Q_0.75_).

^a^The post-hoc test didn’t show any significant differences for HbA1c.

Apart from this the total cholesterol as well as its LDL- and HDL-fraction increased as expected under a high fat diet, which also indicated a high adherence to the dietary targets.

### Changes of lipids under HFD

**Figure 1:**
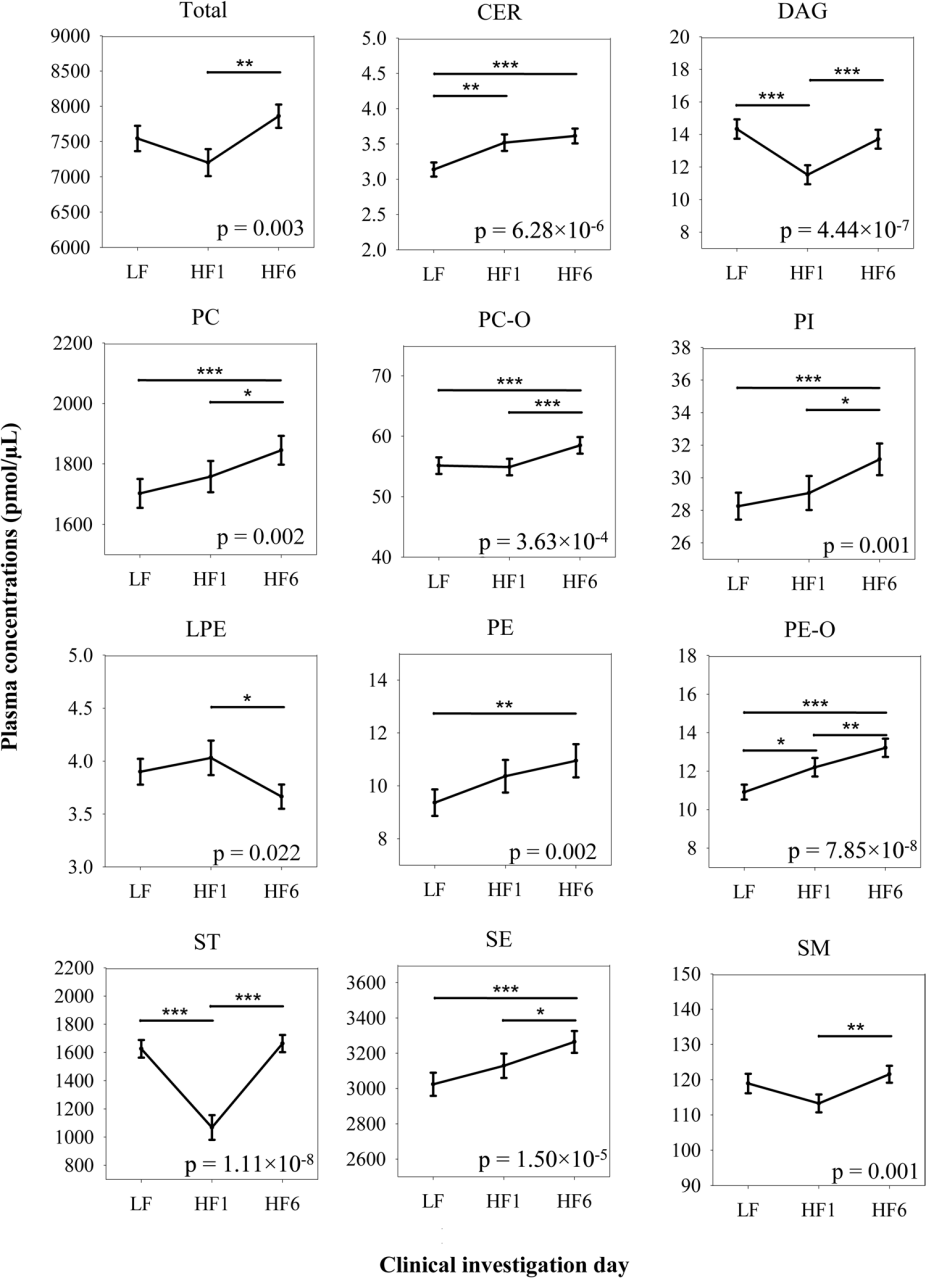
Changes in plasma concentration of the different lipid classes. After LF the classes reacted differently with a monotonous reaction, a time dependent increase or decrease or a counter regulation. The stable lipid classes (TAG and LPC, Supplementary Figure S1) are not shown here. (rep.M. ANOVA + Bonferroni adjustment; *p < 0.05, **p < 0.01, ***p < 0.001, mean ± SEM).

The majority of lipid classes, except LPCs and TAGs (Supplementary Figure S1), changed significantly during the study. The total lipid concentration in plasma increasing between HF1 and HF6 (p = 0.003, rep.M. ANOVA). After LF we observed five different types of reactions:

1. A monotonous increase (PE-Os, PEs, p_PE-Os_ = 7.85 × 10^−8^, p_PEs_ = 0.002),
2. An acute accumulation within one week HFD with a statistically stable plateau afterwards (CERs, p_CERs_ = 6.28 × 10^−6^),
3. A stable concentration to HF1 with a delayed reaction in both directions afterwards (increase: PCs, PC-Os, PIs, SEs and SMs, p_PCs_ = 0.002, p_PC-Os_ = 3.63 × 10^−4^, p_PIs_ = 0.001, p_SEs_ = 1.50 × 10^−5^, p_SMs_ = 0.001; decrease: LPEs, p_LPEs_ = 0.022),
4. A counter regulation of the change occurring after 1 week after additional 5 weeks (STs and DAGs, p_STs_ = 1.11 × 10^−8^, p_DAGs_ = 4.44 × 10^−7^) and
5. No reaction at all with statistically stable concentrations during the HFD (LPCs and TAGs, p_LPCs_ = 0.348, p_TAGs_ = 0.119) (Fig. 1).

Although the classes as an entirety showed a specific response pattern in their total concentrations, changes in the composition of individual lipid species occurred, too.

The concentrations of 102 of the 150 lipid species (68%) changed significantly during the study (0.044 ≥ p ≥ 5.63 × 10^−15^, rep.M. ANOVA; Supplementary Figure S2). 13 of them reacted thereby monotonously (3 decreased, 10 increased), whereas the majority of species responded acutely between LF and HF1 (n = 45; 23 increased, 22 decreased) or in a delayed-fashion from HF1 to HF6 (n = 31; 30 increased, 1 decreased). The remaining 13 species responded more adaptively with a counter regulation, with 11 decreasing at first and then increasing again. The 42 species, which did not change their concentrations at all, belonged to 8 different classes and consisted of more than half of the measured TAGs as well as half of the LPEs and nearly a third of the measured PCs and PC-Os.

Our attempts to classify or cluster the different changing lipid species were not successful. Neither the head groups nor the degree of saturation seemed to determine the reaction pattern of the species. This is also true for the length or the presence of specific fatty acids. The degrees of saturation in the different lipid species did not show global response patterns despite of the high fat diet.

Nevertheless, the lipidome showed a significant change in flexibility (n = 150; p = 1.72 × 10^−7^, rep. M. ANOVA; Supplementary Figure S3). After a significant increase during the first week of HFD the median flexibility of the lipid species decreased again and reached the initial value (LF: 0.22 [0.18, 0.28] vs. HF1: 0.24 [0.19, 0.33] vs. HF6: 0.23 [0.19, 0.30]).

### Impacts on lipid concentrations

We next analysed the impact of sex, BMI and age (linear and quadratic) by the fixed factors of linear mixed modelling on lipid concentration for the cumulative lipid classes as well as for the lipid species. The latter enabled us to look for deviations of specific species concentrations compared with their class.

6 of the 13 classes as well as the total concentration of lipids were sex dependent, with a decrease in five classes in male (SEs, p_SEs_ = 4.00 × 10^−4^ and PCs, PEs, PIs, SMs, all p < 1 × 10^−16^) and a single class in female (LPCs, p < 1 × 10^−16^) participants, respectively. Total concentration was also lower in male participants (p_Total_ = 0.002).

**Figure 2:**
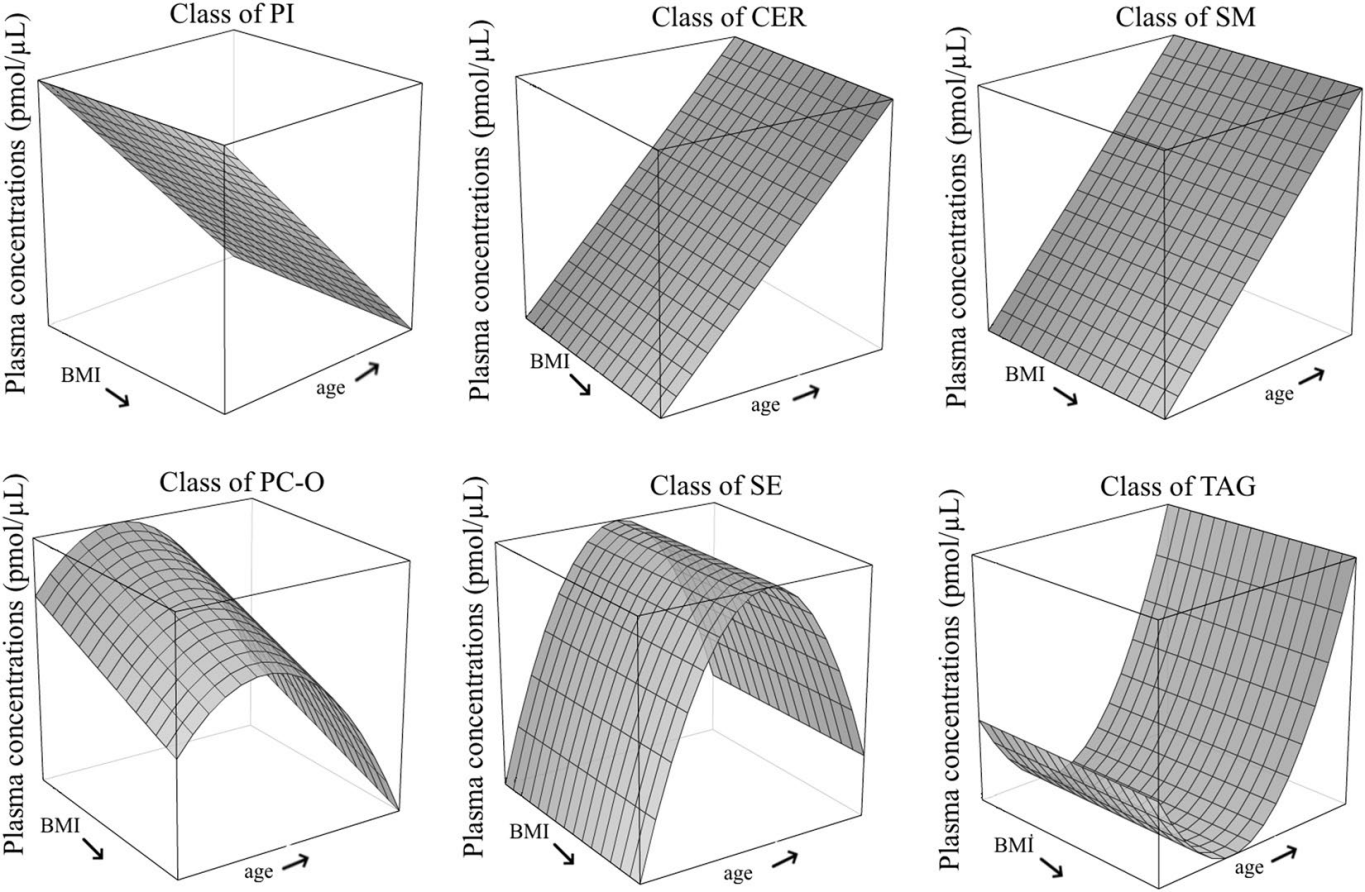
Effects of BMI and age on plasma concentrations of lipid classes. The mesh plots show the interaction of BMI and age based on the fixed factors of the linear mixed models with the plasma concentration of the lipid class on the z axis. The axes ranged from 18 to 80 years (age) and 17.5 to 30 kg/m^2^ (BMI). Each mesh covers 4.13 years and 0.83 kg/m^2^. Not shown here are classes, which were not influenced by at least one of the factors.

In contrast BMI seemed to only affect the phosphatidylcholine ethers at class level by increased concentrations in leaner participants (PC-Os: β_BMI_ = −1.44, p_BMI_ = 0.002; Fig. 2).

CERs, SMs and STs (p_CERs_ = 6.00 × 10^−4^, p_SMs_ = 0.009, p_STs_ = 0.008) showed also significantly lower concentrations in younger participants, whereas PIs seemed to decrease with higher age (all p_PIs_ = 0.011). In addition to their linear age dependency PC-Os, SEs and TAGs had quadratic effects of age with opposite direction (PC-Os: β_age_ = 2.16, p_age_ < 1 × 10^−16^ vs. ${{\rm{\beta }}}_{{{\rm{age}}}^{2}}$ = −0.03, ${{\rm{p}}}_{{{\rm{age}}}^{2}}$ < 1 × 10^−16^; SEs: β_age_ = 65.36, p_age_ = 0.001 vs. ${{\rm{\beta }}}_{{{\rm{age}}}^{2}}$ = −0.64, ${{\rm{p}}}_{{{\rm{age}}}^{2}}$ = 0.011; TAGs: β_age_ = −33.78, p_age_ = 0.026 vs. ${{\rm{\beta }}}_{{{\rm{age}}}^{2}}$ = 0.41, ${{\rm{p}}}_{{{\rm{age}}}^{2}}$ = 0.029) indicating that the classes reacted differently in younger and older participants. The TAGs were negatively age-dependent until an age of 40 to 41 and then increased with increasing age again, whereas the PC-Os and SEs had their highest concentrations at an age of 40 to 41 and 51, respectively. The total concentration showed also a linear and quadratic effect of age, with the concentrations increasing until an age of 47 and decreasing again afterwards (Total: β_age_ = 137.61, p_age_ = 0.012 vs. ${{\rm{\beta }}}_{{{\rm{age}}}^{2}}$ = −1.45, ${{\rm{p}}}_{{{\rm{age}}}^{2}}$ = 0.033).

Surprisingly the polynomial effect of age for the classes of PC-Os and SEs seemed to be driven by only a very small number of species or might be a synergetic effect. PC-O [36:5], SE [45:3, (27:1, 18:2)] and SE [47:5, (27:1, 20:4)] were the only species with significant age and age^2^ effects (PC-O_[36:5]_: β_age_ = 0.45, p_age_ < 1 × 10^−16^ vs. ${{\rm{\beta }}}_{{{\rm{age}}}^{2}}$ = −0.01, ${{\rm{p}}}_{{{\rm{age}}}^{2}}$ < 1 × 10^−16^; SE_[45:3, (27:1, 18:2)]_: β_age_ = 41.18, p_age_ = 8.00 × 10^−4^ vs. ${{\rm{\beta }}}_{{{\rm{age}}}^{2}}$ = −0.41, ${{\rm{p}}}_{{{\rm{age}}}^{2}}$ = 0.008; SE_[47:5, (27:1, 20:4)]_: β_age_ = 8.88, p_age_ = 4.00 × 10^−4^ vs. ${{\rm{\beta }}}_{{{\rm{age}}}^{2}}$ = −0.10, ${{\rm{p}}}_{{{\rm{age}}}^{2}}$ = 0.001) with the turning point at the age of 42, 51 and 44, respectively. In contrast none of the TAG species had a significant quadratic age effect and only two of them were age dependent (TAG_[52:4]_: β_age_ = −0.69, p_age_ = 0.017, TAG_[54:4]_: β_age_ = −0.39, p_age_ = 0.016), which indicates a synergetic effect.

**Figure 3:**
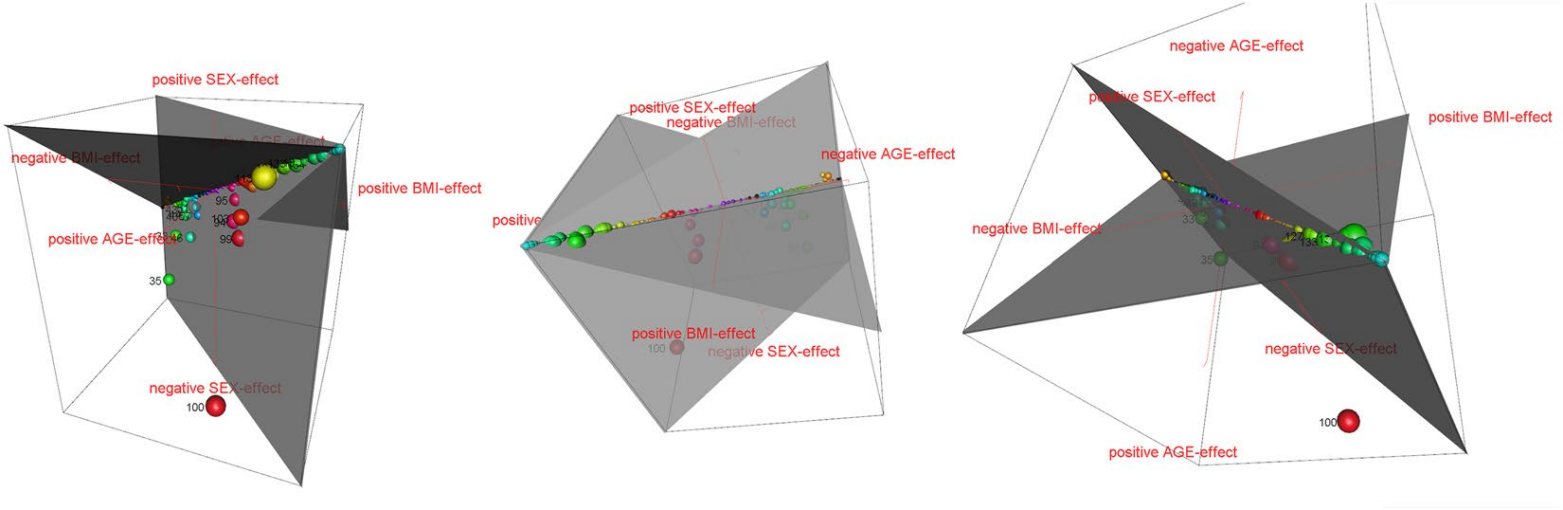
Effects of BMI, age and sex on plasma concentrations of lipid species. The 3D bubble plot shows the interaction of BMI, age and sex based on the fixed factors of the linear mixed models with young women with low BMI being the reference. The size of a bubble represents the median concentration at LF. The numbering of the species has been restricted to those with a deviation from the principal diagonal. The colours also represent the different lipid species. The deviation of the bubbles from the principal diagonal represents the effect of the respective species on the parameter where the bubble is deviated to. The species were sorted alphabetically with rising chain length and the plot is shown here in three perspectives. (See also Media S1).

When the data was analysed at species level (Fig. 3), 25 of the 150 lipid species showed a significant age dependency (p < 0.04 to p < 1 × 10^−16^), with 24 being members of also age dependent classes including four CERs and ten SEs. Thereby, the species effects always mirrored the class effect without exception. PC [36:5, (16:0, 20:5)] was the only age affected species (β_age_ = 0.14, p_age_ = 3.00 × 10^−4^) belonging to an age independent class and increased in older participants.

In conclusion, age played a minor role in contrast to BMI effects and sex differences. Although only PC-Os seemed to be BMI dependent on class level, 44 of the 150 species were significantly affected by the participants’ BMI (p < 0.042 to p < 1 × 10^−16^), with 22 decreasing and 22 increasing by increased BMI. Interestingly, all negatively associated lipid species belonged to classes of phospholipids and *vice versa* only two phosphatidylcholines with higher carbon atom amount (n_C atoms_ ≥ 38) were positively associated with BMI. The 20 remaining increased lipid species were members of SE (3 of 14) and TAG (17 of 32).

75 of the 150 lipid species were significantly sex dependent (p < 0.05 to p < 1 × 10^−16^). 8 of the 75 species showed an unexpected sexual dimorphism, as their class overall did not have a significant sex dependency. These 8 species belonged to LPE and PC-O, and a single TAG, with concentrations of the LPEs as well as one of the PC-Os being higher in male participants and the concentrations of the remaining two PC-Os and the TAG being higher in female participants. The other 67 species mirrored the sex dependency of their class concentration and were decreased in men.

Nevertheless, the intercept, which was significant for all classes and 80% of the species, suggests that there are more influencing factors, which we have not considered in our model, yet.

### Heritability of lipid concentrations

**Figure 4:**
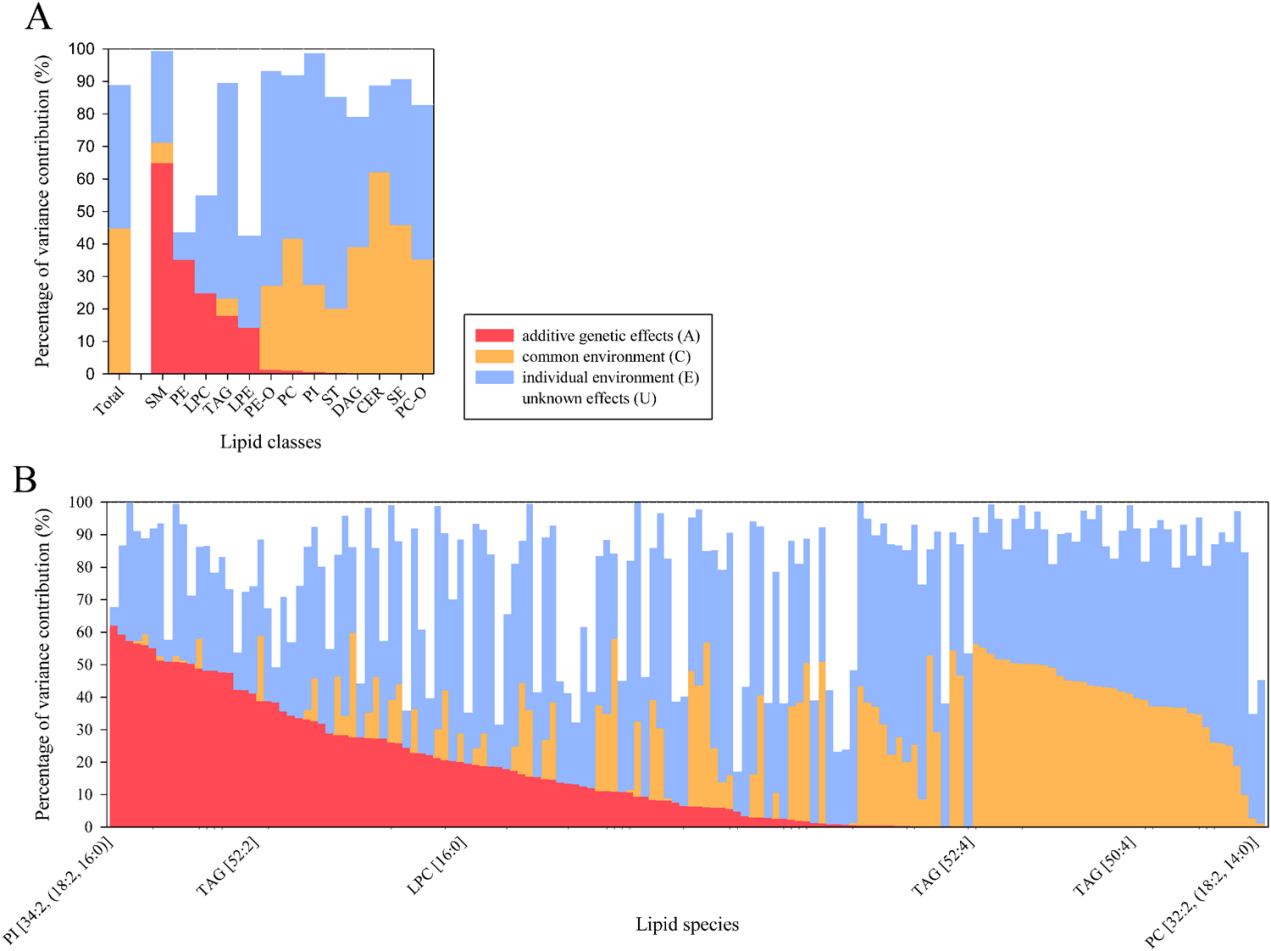
Percentage of variance contribution determined by a linear mixed model based on the repeated measured ACE model (LF, HF1, HF6) for lipid classes (**A**) and lipid species (**B**). The parameters were sorted/ranked by the rounded values for heritability (colour red), common environment (colour orange), individual environment (colour blue) and unknown effects (colour white). (See also Tables S1 and S2).

The twin design of the NUGAT study enabled us to estimate the heritability of the different lipid classes and species. The linear mixed model added a fourth component representing unknown effects, which could not be described by the classic ACE model. In this way the model reveals a wide range of heritability in lipid classes and single species up to 65% and 62% respectively (Fig. 4).

Following our definition of a high heritability (A-value > 0.4), only the class of SM were mainly determined by additive genetic effects (A_SM_ = 0.650), with the remainder of the variance mainly driven by individual environment (E_SM_ = 0.282) (Table S1).

LPC and PE had a moderate heritability between 25 and 35%, respectively. The remaining classes seemed to be more environmentally determined, with SE, CER, and PC-O showing no heritability at all. Nevertheless, three of the classes also showed very high proportions of unknown effects (U_LPC_ = 0.449, U_PE_ = 0.562, U_LPE_ = 0.574), containing all lysophosholipid classes.

The analysis on lipid species level again reveals a remarkably more complex pattern than the analysis on class level (Table S2). 19 of the 150 lipid species had high A-values (>0.4) and were therefore highly heritable. Surprisingly, the lipid with the highest A-value (PI [34:2, (18:2, 16:0)]; A_PI [34:2, (18:2, 16:0)]_ = 0.621) did not belong to a heritable class (A_PI_ = 0.007). Additionally, 16 of the 19 highly heritable lipid species were phospholipids, including both measured phosphatidylethanolamines.

In contrast 38 lipid species did not show any heritability, including 7 (50%) of the SEs as well as 15 (47%) TAGs.

33 of the 150 species (22%) showed also a high impact of the unknown effects up to almost 83% of the observed variance (U_PC-O [32:0, (18:0, 14:0)]_ = 0.827).

### Heritability of changes in lipid concentrations

After using the ACE model to calculate A,C and E for the changes from LF to HF1, HF1 to HF6 and LF to HF6, we could detect changes, which were highly heritable across all three measurements, for only one species – PE [38:4, (20:4, 18:0)] (increased significantly between LF and HF6; c_LF_ = 6.23 ± 0.34 vs. c_HF6_ = 6.78 ± 0.41 and c_HF1_ = 6.59 ± 0.37 pm/µL, p = 0.026; A_ΔLF,HF1_ = 0.54, A_ΔHF1,HF6_ = 0.48, A_ΔLF,HF6_ = 0.53). In contrast to this 113 of the 150 species did not have any notable heritability at all in their concentration changes. The remaining 36 lipids showed only unsteady heritability without class or fatty acid dependency.

## Discussion

Our NUGAT study provides new insights into the relationship of the lipidome and nutritional challenges in humans. Via high throughput quantitative mass spectrometry we were able to depict the variability of the human plasma lipidome on a molecular species level. In addition, we present the first twin study that addresses the heritability of concentrations of human lipid species in blood.

Our major finding was the high heritability of basal concentrations of specific lipid species rather than lipid classes and their strong dependence on sex, BMI and age. This underlined the importance of reliable mass spectrometry-based lipidomics to define their specific roles in predicting disease risks and disease progression.

The high heritability resulted most likely from genetic variance in biosynthetic pathways. Our data therefore provide guidance for targeted searches for genetic variants involved and require further studies. Lipid species are increasingly linked to specific pathologies and the more heritable ones are likely to affect disease risk and progression in specific disease phenotypes. This opens a wide field of investigation to define the predictive and pathophysiological role of lipids in medicine. We observed strong effects of sex in our rather young population. Sex effects may relate to genetic differences in the Y-chromosome, to imprinting or to sex steroid dependent effects. The latter moreover vary in females with the ovarian cycle and most of our female twins were premenopausal. Lipid metabolism differs extensively between males and females including the whole body lipid content, anthropometric lipid distribution particularly in the visceral and subcutaneous compartments, and turnover^17–19^.

We were able to demonstrate a strong sensitivity of changes in specific lipid concentrations already after a single week after the switch to a Western-style high fat diet, confirming the strong impact of diet on the lipidome. Studies relating the lipidome to diseases therefore need detailed dietary information and, if possible, standardized diets.

Unexpectedly, we observed limited heritability of the diet induced changes in lipids despite – or rather because of - the significant effects of the diet. This implies that the reactions of lipid classes to nutrition may be rather conserved among all subjects with little individual variation which would then result in low heritability estimates.

An interesting aspect resulted from the observation that our healthy and mostly young participants were partly able to adapt and deal with the nutritional challenge after additional five weeks. This applied to initial changes after 1 week, which induced an opposite change after 6 weeks suggesting an adaptive response. The increasing and then decreasing flexibility of the lipidome, which indicates a synchronization of the mostly young cohorts’ lipidome after the initial challenge, also supported this observation. This may reflect an important ability to compensate nutritional challenges of an unhealthy diet and may be a reason why high fat diets show relatively modest associations with cardiovascular disease^6^. It is likely, that this flexibility decreases in the presence of metabolic diseases such as obesity, dyslipidaemia or impaired glucose metabolism. In addition, age is likely to play a major role in metabolic flexibility and future studies should address this aspect in more detail. Moreover, the ability or inability to adapt may determine future risks of diet related diseases. Therefore dietary tests may be devised based on the ability to regulate disease related lipid species and these tests should include short term and longer term lipidomic responses.

Importantly, the changes in species not only seemed to be much more complex than the changes in their overall classes; it was impossible to classify the differently reacting species according to their head group, degree of saturation or related chemical properties. A similar diversity was found for the heritability of lipid concentrations. Especially the most heritable lipid species (PI [34:2, (18:2, 16:0)]) belonged to a non-heritable class (A = 0.007), whose other members only reached mostly low or moderate A-values (<0.39). This might be a hint to a central role of this specific species in its lipid class. However, additive genetic effects must be considered for sex, BMI and age.

Limitations of our study apply to the ethnicities since only Caucasian subjects were studied. Furthermore, we included a variance component for unknown effects (U) in our linear mixed models. Possible explanations for this unknown variance might be short-term fluctuations and also technical variability. Also dominant genetic effects, which were set equal to zero in the ACE model, might influence this component as well as other life style factors (e.g. smoking [25 smoker, 12 ex-smoker] or drinking), which we did not regard. Further investigations to clarify this proportion of variance are needed.

Lipidomics requires more characterization and understanding of its complexity, interactions and functions^12, 13^ in particular, with regard to specific and minor lipid classes. For example, a study in Finish twins^4^ described associations between obesity and increasing concentrations of lysophosphatidylcholines, whereas Barber *et al*.^20^ observed the opposite with decreased concentrations of LPCs in obesity and Type II diabetes. Our data suggest that it is necessary to distinguish between different species as the overall concentrations of lipid classes were not informative. Additionally, Ishikawa *et al*.^17^ showed, that not only age and sex altered the lipid profile, but also the interaction of both factors.

There is a scientific consensus that concentrations of lipoproteins such as HDL and LDL are highly heritable in humans and associated with disease risks^21–23^. However there are only few studies addressing the heritability of the lipid profile itself.

Scheitz *et al*.^12^ could detect that most lipid classes were highly heritable in 92 inbred lines of *D*. *melanogaster*, although especially PEs and PCs had species with a greater variability in the proportions of additive genetic effects. Apart from the degree of saturation, the ratio of PCs to PEs – as main structural membrane components - is a crucial regulator for membrane fluidity and therefore equally important for homeoviscous and thermal adaptation in poikilothermic organisms^12, 24, 25^. Homoeothermic organisms, like humans, do also alter their membrane fluidity to control e.g. permeability and activity of membrane proteins, which might be an explanation for the great variability of heritable and non-heritable species of PCs and PEs, which we could confirm.

Interestingly, the great variability of heritable and non-heritable species seemed to occur also in other subsets of human plasma metabolomics. Liu *et al*.^26^ observed a heritability pattern for 342 plasma proteins in their twin population with apolipoprotein (a) having the highest heritability, which looks quite similar to our heritability pattern of the plasma lipidomics.

Obviously, it is of importance to control for confounders such as weight/BMI, age and life style factors, particularly nutrition. Only the understanding of the characteristics of a healthy lipid profile will lead to the ability to investigate lipidomics in diseases. Therefore, the next steps in the NUGAT study will be to clarify the function and interaction of lipid species and interdependencies with genomic and metabolic data in our healthy participants.

## Material and Methods

### Participants

The *NutriGenomic Analysis in Twins* (NUGAT) study involved a cohort of 92 participants (46 pairs of twins – 34 monozygotic and 12 dizygotic), 58 female and 34 male. The age ranged from 18 to 70 with a median of 25. Only twins with a Body Mass Index (BMI = weight [kg]/height [m] × height [m]) between 18 and 30 kg/m^2^ were included. Physiological data of the participants at Screening are summarized in Table 1.

### Study Design

The NUGAT study was registered at www.clinicaltrials.gov (June 1, 2012, NCT01631123). The study protocol was approved by the local Ethical Committee of the Charité University of Medicine, Berlin, Germany, and was in accordance with the Declaration of Helsinki of 1975, as revised in 1983. All participants gave written informed consent prior to the study.

**Figure 5:**
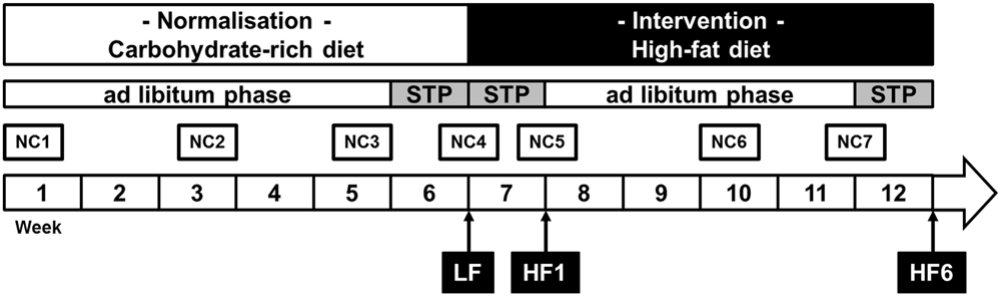
Study design. The participants performed two isocaloric diet periods. During the first six weeks they received a low-fat, carbohydrate-rich diet as standardization for their eating behaviours. After a first clinical investigation day (LF) the participants switched to a high-fat, low-carbohydrate diet and underwent two more CIDs after one (HF1) and additional five weeks (HF6) on the high-fat diet. During the week before a clinical investigation day the participants received detailed meal plans for each day to ensure a standardized dietary pattern (STP) and at least every two weeks nutritional counselling (NC) took place.

Aim of the study was to investigate the metabolic response to a nutritional intervention consisting of an isocaloric shift from a low fat to a high fat diet. Additionally, the study was designed as a twin study to examine different (nutri-) genetic factors e.g. the heritability of selected biomarkers in this context. A time line of the study is shown in Fig. 5.

The pairs of twins, recruited from a twin register (HealthTwiSt GmbH, Berlin, Germany) and by public advertisements from the general population of Berlin and Brandenburg in Germany, arrived at the metabolic research unit at the German Institute for Human Nutrition for a baseline measurement (screening). The participants started the study with a six weeks period of carbohydrate-rich, low fat diet (30E% fat, 55E% carbohydrates, 15E% protein), which we used to standardize the nutritional behaviour of our participants. After the standardization they switched to six weeks of a low carbohydrate, high fat diet (45E% fat, 40E% carbohydrates, 15E% protein). Both diets were adjusted in accordance with the individual energy requirements of the participants and were composed isocaloric.

During the last week of the carbohydrate-rich low fat diet as well as the first and sixth week of the low carbohydrate high fat diet approximately 70% of the food was provided and the participants received detailed meal plans for each day to ensure a standardized dietary pattern.

The investigation was carried out over three different days as follows: The first clinical investigation day (LF) was performed directly at the end of the normalization period. The second investigation day (HF1) was set after one week on the high fat diet and the third (HF6) after additional five weeks on the HFD, to assess not only the short-term but also the long-term effects of the higher fat intake.

### Mass spectrometry lipidomics

Mass spectrometry-based lipid analysis was performed at Lipotype GmbH (Dresden, Germany) as described elsewhere^27^. Plasma lipids were extracted with methyl tert-butyl ether/methanol (7:2, V:V) as in Matyash *et al*.^28^. Plasma was diluted 50× with 150 mM ammonium bicarbonate (in water). For lipid extraction an equivalent of 1 µL of undiluted plasma was used. Internal standards were pre-mixed with the organic solvents mixture. The internal standard mixture contained (per one extracted sample): 1 nmol cholesterol D6, 100 pmol cholesterol ester 20:0 (SE), 50 pmol ceramide 18:1;2/17:0 (CER), 100 pmol diacylglycerol 17:0/17:0 (DAG),500 pmol phosphatidylcholine 17:0/17:0 (PC), 50 pmol phosphatidylethanolamine 17:0/17:0 (PE), 50 pmol phosphatidylinositol 16:0/16:0 (PI), 50 pmol lysophosphatidylcholine 12:0, (LPC) 30 pmol lysophosphatidylethanolamine 17:1 (LPE), 50 pmol triacylglycerol 17:0/17:0/17:0 (TAG) and 200 pmol sphingomyelin 18:1;2/12:0 (SM). After extraction, the organic phase was transferred to an infusion plate and dried in a speed vacuum concentrator. Dried extract was re-suspended in 7.5 mM ammonium acetate in chloroform/methanol/propanol (1:2:4, V:V:V). All liquid handling steps were performed using Hamilton Robotics STARlet robotic platform with the Anti Droplet Control feature for organic solvents pipetting.

Samples were analysed by direct infusion in a QExactive mass spectrometer (Thermo Scientific) equipped with a TriVersa NanoMate ion source (Advion Biosciences). Samples were analysed in both positive and negative ion modes with a resolution of Rm/z = 200 = 280000 for MS and Rm/z = 200 = 17500 for MSMS experiments, in a single acquisition. MSMS was triggered by an inclusion list encompassing corresponding MS mass ranges scanned in 1 Da increments. Both MS and MSMS data were combined to monitor CE, DAG and TAG ions as ammonium adducts; PC, PC O-, as acetate adducts; and PE, PE O- and PI as deprotonated anions. MS only was used to monitor LPE as deprotonated anion; CER, SM and LPC as acetate adducts and cholesterol as ammonium adduct.

Data were analysed with in-house developed lipid identification software based on LipidXplorer^29, 30^. Data post-processing and normalization were performed using an in-house developed data management system. Only lipid identifications with measured mass deviations below 3 ppm from the theoretical mass for MS and 8 ppm for MSMS peaks, a signal-to-noise ratio >5, and a signal intensity 5-fold higher than in corresponding blank samples were considered for further data analysis.

With this method 150 lipid species from 13 classes (Supplementary Figure S4) were measured in 246 fasted blood samples on each clinical investigation day. The batch corrected dataset spanned all major lipid classes including the information on fatty acids of different degrees of saturation and various odd and even carbon chain lengths (Table 3).

**Table 3:** Characteristics of the measured lipidomes.

Class name	Abbreviation	LF [pmol/µL]	Species	range of species
		mean ± SEM	n	
ceramide	CER	3.14 ± 0.10	5	[38:1] to [42:2]
phosphatidylcholine	PC	1702.45 ± 47.71	34	[30:0] to [38:6]
phosphatidylcholine ether	PC-O	55.13 ± 1.38	20	[32:0] to [38:5]
lysophosphatidylcholine	LPC	83.04 ± 2.86	9	[14:0] to [22:6]
phosphatidylethanolamine	PE	9.36 ± 0.50	2	[36:2] to [38:4]
phosphatidylethanolamine ether	PE-O	10.91 ± 0.39	5	[34:3] to [38:6]
lysophosphatidylethanolamine	LPE	3.90 ± 0.12	6	[16:0] to [22:6]
phosphatidylinositol	PI	28.26 ± 0.83	7	[34:1] to [38:4]
diacylglyceride	DAG	14.33 ± 0.60	3	[34:1] to [36:3]
triacylglyceride	TAG	1017.90 ± 41.72	32	[46:1] to [56:7]
sphingomyeline	SM	118.92 ± 2.76	12	[14:0] to [24:1]
sterolester	SE	3023.85 ± 65.93	14	[41:1] to [49:7]
sterol	ST	1625.57 ± 62.98	1	[27:1]
Total	—	7543.59 ± 179.08	150	—

The lipidomic data is open accessible as Supplementary Material.

### Other measurements

Routine serum and fasted blood parameters were measured using standard techniques (ABX Pentra 4000, HORIBA ABX SAS, Montpellier, France). Low density lipoprotein cholesterol concentrations were calculated using the Friedewald formula^31^.

### Statistical analysis

Before the data was released for analysis, an analysis of plausibility was performed. Unusual values that were outside of 3-fold interquartile range (IQR) were declared as extreme outliers and were not considered in further analysis.

For the study of heritability, the ACE model for *A*dditive genetics, *C*ommon and unique *E*nvironment was applied for every clinical investigation day. This is a model based on a covariance analysis of the mono- and dizygotic twin pairs, in which the variance is determined that can be traced back to additive genetics (a^2^), common environment (c^2^) and individual environment (e^2^) of the twins^32^. A more detailed description of the structural equation models (including ACE model) can be found in the Supplementary Material. The ACE model was also checked against the simpler AE model based on a likelihood ratio test and replaced if necessary.

For each lipid class and for each lipid species, we fitted the linear mixed-effects model^33^:

$$\begin{array}{rcl}{{\rm{y}}}_{{\rm{ij}}} & = & {{\rm{\beta }}}_{0}+{{\rm{\beta }}}_{{\rm{sex}}}{{\rm{x}}}_{{\rm{ij}}}^{({\rm{sex}})}+{{\rm{\beta }}}_{{\rm{BMI}}}{{\rm{x}}}_{{\rm{ij}}}^{({\rm{BMI}})}+{{\rm{\beta }}}_{{\rm{age}}}{{\rm{x}}}_{{\rm{ij}}}^{({\rm{age}})}+{{\rm{\beta }}}_{{{\rm{age}}}^{2}}{{\rm{x}}}_{{\rm{ij}}}^{({{\rm{age}}}^{2})}\\  &  & +{{\rm{\beta }}}_{{\rm{CID}}}{{\rm{x}}}_{{\rm{ij}}}^{({\rm{CID}})}\,+{{\rm{u}}}_{{{\rm{a}}}^{2}{\rm{j}}}{{\rm{z}}}_{{\rm{ij}}}^{({{\rm{a}}}^{2})}+{{\rm{u}}}_{{{\rm{c}}}^{2}{\rm{j}}}{{\rm{z}}}_{{\rm{ij}}}^{({{\rm{c}}}^{2})}+{{\rm{u}}}_{{{\rm{e}}}^{2}{\rm{j}}}{{\rm{z}}}_{{\rm{ij}}}^{({{\rm{e}}}^{2})}+{\epsilon }_{{\rm{ij}}}\end{array}$$

with fixed factors for sex, BMI, age, age^2^ and clinical investigation day (CID, representing the diet) and random effects for the repeated measured ACE model, with j ∈ (1, …, 92) as subject index and i ∈ (0, …, 3) as CID index. On this occasion the age effect was considered with two factors, a linear and a quadratic, because it is well known, that age effects were not linear in most cases. Both terms allowed describing parabolic interactions between age and the dependent variable and identifying the turning point. Akaike’s information criterion (AIC) was used to find the best fitted model.

Heritability was defined as ${\rm{A}}={{\rm{\sigma }}}_{{{\rm{a}}}^{2}}^{2}/{{\rm{\sigma }}}_{{\rm{total}}}^{2}$ with ${{\rm{\sigma }}}_{{\rm{total}}}^{2}={{\rm{\sigma }}}_{{{\rm{a}}}^{2}}^{2}+{{\rm{\sigma }}}_{{{\rm{c}}}^{2}}^{2}+{{\rm{\sigma }}}_{{{\rm{e}}}^{2}}^{2}+{{\rm{\sigma }}}_{\varepsilon }^{2}$, and the rest of the variance components were defined in the same way with C for the common environment, E for the individual environment and U for the variance, we could not explain in the model represented by the residuals. An A-value greater than 0.40 was defined as high heritability and used as threshold.

To investigate the changes of the lipids a single factor univariate analysis of variance (ANOVA) with repeated measures (rep.M.) and Bonferroni adjustment was used. The requirements for the ANOVA were tested by the Shapiro-Wilk-test and Mauchly’s sphericity-test with ln- and/or Greenhouse-Geisser transformation if necessary.

The flexibility of a lipid was defined as the quartiles dispersion coefficient (QDC) of the CID dependent lipid concentrations, which is calculated as:

$${{\rm{QDC}}}_{{\rm{Lipid}},{\rm{CID}}}={[\frac{0.5({{\rm{Quartile}}}_{0.75}-{{\rm{Quartile}}}_{0.25})}{0.5({{\rm{Quartile}}}_{0.75}+{{\rm{Quartile}}}_{0.25})}]}_{{\rm{Lipid}},{\rm{CID}}}.$$

Unless otherwise stated, a significance level of α = 0.05 was used. If the global significance level had to be adjusted due to multiple testing, the Benjamini-Hochberg method was used. All mean values are given as mean ± SEM, all median values as median, [Q_0.25_, Q_0.75_] (=IQR_0.25, 0.75_).

The calculations were performed with IBM SPSS Statistics (version 20) and the integrated development environment RStudio (version 0.98.1091), which is based on R (and 3.1.2). The ACE model was executed in an older version of R (version 2.1.5), since the necessary package OpenMX was not upward compatible. Graphs were designed in Sigma Plot (version 11).

## Electronic supplementary material

Supplementary Material

Media S1

Dataset 1
