# Supplementary material for: Heritability and responses to high fat diet of plasma lipidomics in a twin study
Source: Sci Rep. 2017 Jun 16;7:3750. doi: 10.1038/s41598-017-03965-6 (PMC5473837; doi:10.1038/s41598-017-03965-6)

Heritability and responses to high fat diet of plasma lipidomics in a twin study.

Turid Frahnow*; Martin A Osterhoff, Dr.; Silke Hornemann; Michael Kruse, Dr.; Michal A Surma, Dr.;

Christian Klose, Dr.; Kai Simons, Prof.Dr.; Andreas FH Pfeiffer, Prof.Dr.

Structural equation model

Structural equations models (including ACE model) are used in epidemiology and (behavior) genetic research to reveal the importance for environmental and genetic effects on phenotypes in health and disease. They are used in Twin studies but were also generalized to Case-Control Family Data[1,2].
In Twin studies, the models are mainly based on three assumptions:

1. Monozygotic twins (MZ) develop from a single zygote and share all of their alleles. Therefore they are nearly genetically identical.
2. Dizygotic twins (DZ) develop from two zygotes and share on average 50% of their alleles like non-twin siblings.
3. MZ and DZ share mainly the same environment.

These assumptions lead to the ability to separate genetic and environmental effects by comparing the within-pair (dis-)concordance between monozygotic and dizygotic twin pairs. For example, if monozygotic twins show a high concordance (or only small within-pair variance/variation) in a trait, whereas dizygotic twins vary much more, it is more likely that this trait is driven by genetic effects. In contrast, if monozygotic and dizygotic twins only show slight variance within the pairs for the same trait, it is most likely, that the trait is mostly driven by a within-pair shared environment of the twins.

Usually the structural equation models are able to identify one of two genetic factors:

- Additive genetic effects (A),
- Dominant or non-additive genetic effects (D),

and up to two environmental factors:

- The shared environment or common environment (C), that have the effect of making siblings more similar to one another (e.g. shared family structure, place of birth/living, nutrition in childhood),
- The individual environment (E), that have the effect of making siblings unique (e.g. smoker status, nutrition in adulthood).

Nevertheless, the discrimination between shared and individual environment effectors in terms of biology is difficult.

1 Javaras, K. N., Hudson, J. I. & Laird, N. M. Fitting ACE structural equation models to case-control family data. *Genet Epidemiol* **34**, 238-245, doi:10.1002/gepi.20454 (2010).
2 Maes, H. H. in *Encyclopedia of Statistics in Behavioral Science* (John Wiley & Sons, Ltd, 2005).

Supplementary figures

Supplementary Figure S1: Plasma concentration of the stable lipid classes after LF. In contrast to the concentrations of the other classes, the concentrations of TAG and LPC did not change significantly. (rep.M. ANOVA + Bonferroni adjustment; n.s. – not significant, mean ± SEM)

Supplementary Figure S2: Pie charts of class dependent species changes after normalization (LF, HF1, HF6). Although the overall concentration of some classes (like LPC and TAG) did not change, single species reacted to the nutritional challenge of the HFD. Moreover the single species showed an obviously more complex reaction pattern than the overall concentration, which necessitates the distinction of species. (rep.M. ANOVA + Bonferroni adjustment)

Supplementary Figure S3: Flexibility of lipid species (n=150). The flexibility of the lipidome depicted the ability of the participants to adapt to the HFD. The increase after one week HFD vanished completely after additional five weeks and the participants were synchronized again by lowering the variability within the mostly young cohort. The flexibility is shown as the quartiles dispersion coefficient for each CID and each lipid species. The triangles represented the arithmetic mean of the lipid species. (rep.M. ANOVA + Bonferroni adjustment)

Supplementary Figure S4: Flow chart for the analysis of lipid species. We first excluded the samples, which did not pass the quality control (n=30), then the lipid species, which were only measurable in < 80% of the remaining samples. We included 150 lipid species measured in 246 samples in the analysis.

Media S1: Interactive 3D bubble plot (Fig.5) with R script and data. (3DBubble_small.mov, Bubbleplot.R, Lipidomics_150_corrected_diagonale.csv)

Supplementary Table S1: Absolute amount of variance contribution determined by a linear mixed model based on the repeated measured ACE model (LF, HF1, HF6) for lipid classes. The classes showed a high variability for additive genetic effects (heritability). Only SM were highly heritable with A-values greater than 0.40 (= 40%). The parameters – except Total concentration - were sorted/ranked by the rounded values for heritability (A), common environment (C), individual environment (E) and unknown effects (U).

	**A**	**C**	**E**	**U**
**Total**	0.005	0.444	0.442	0.108
**SM**	0.650	0.063	0.282	0.004
**PE**	0.353	0.000	0.084	0.562
**LPC**	0.250	0.000	0.301	0.449
**TAG**	0.180	0.053	0.664	0.103
**LPE**	0.144	0.000	0.283	0.574
**PE-O**	0.014	0.259	0.661	0.067
**PC**	0.011	0.406	0.504	0.079
**PI**	0.007	0.268	0.713	0.011
**ST**	0.005	0.197	0.652	0.146
**DAG**	0.002	0.390	0.401	0.207
**CER**	0.000	0.621	0.268	0.111
**SE**	0.000	0.460	0.448	0.092
**PC-O**	0.000	0.354	0.475	0.171

Supplementary Table S2: Absolute amount of variance contribution determined by a linear mixed model based on the repeated measured ACE model (LF, HF1, HF6) for lipid species. The species showed a high variability for additive genetic effects (heritability) with values between 0 and 62 %. The parameters were sorted/ranked by the rounded values for heritability (A), common environment (C), individual environment (E) and unknown effects (U).

	A	C	E	U
**PI [34:2, (18:2, 16:0)]**	0.621	0.000	0.058	0.321
**PE [36:2, (18:2, 18:0)]**	0.594	0.000	0.275	0.131
**PC-O [32:0, (16:0, 16:0)]**	0.575	0.000	0.423	0.003
**PC-O [34:3, (16:1, 18:2)]**	0.567	0.008	0.338	0.087
**PE-O [36:5, (16:1, 20:4)]**	0.561	0.035	0.295	0.109
**PC [33:1, (17:1, 16:0)]**	0.552	0.000	0.368	0.081
**SM [36:1]**	0.514	0.014	0.408	0.064
**PE [38:4, (20:4, 18:0)]**	0.511	0.000	0.068	0.421
**PI [36:2, (18:0, 18:2)]**	0.510	0.019	0.467	0.003
**PC-O [36:3, (18:1, 18:2)]**	0.508	0.007	0.419	0.066
**PC-O [34:1, (18:1, 16:0)]**	0.504	0.000	0.210	0.287
**SE [47:4, (27:1, 20:3)]**	0.490	0.091	0.283	0.136
**LPE [16:0]**	0.483	0.000	0.384	0.133
**PC-O [34:1, (16:0, 18:1)]**	0.483	0.000	0.302	0.215
**LPC [18:1]**	0.478	0.000	0.355	0.166
**SM [40:1]**	0.477	0.000	0.257	0.266
**LPE [18:1]**	0.424	0.000	0.115	0.461
**PC [38:4, (20:3, 18:1)]**	0.423	0.000	0.303	0.274
**LPC [20:4]**	0.413	0.000	0.330	0.257

**TAG [52:2]**	0.389	0.202	0.296	0.113
**LPC [18:0]**	0.389	0.000	0.286	0.325
**PI [36:1, (18:0, 18:1)]**	0.386	0.000	0.108	0.507
**PC [38:3, (18:0, 20:3)]**	0.358	0.000	0.352	0.291
**LPE [18:0]**	0.344	0.000	0.227	0.428
**PC [36:3, (20:3, 16:0)]**	0.337	0.000	0.407	0.256
**SM [38:1]**	0.333	0.029	0.502	0.136
**SM [36:2:b]**	0.328	0.132	0.466	0.074
**PI [38:3, (18:0, 20:3)]**	0.320	0.000	0.483	0.197
**PC [36:1, (18:0, 18:1)]**	0.290	0.000	0.260	0.450
**TAG [49:2]**	0.285	0.180	0.375	0.160
**PC [36:2, (20:2, 16:0)]**	0.285	0.058	0.616	0.042
**TAG [56:6]**	0.279	0.321	0.263	0.136
**LPE [20:4]**	0.278	0.000	0.166	0.555
**PC [33:2, (18:2, 15:0)]**	0.276	0.078	0.631	0.015
**SM [36:2:a]**	0.274	0.189	0.397	0.139
**DAG [34:1, (16:0, 18:1)]**	0.274	0.000	0.301	0.425
**PC [38:6, (22:6, 16:0)]**	0.262	0.132	0.599	0.007
**TAG [54:6]**	0.260	0.181	0.440	0.118
**LPC [20:3]**	0.245	0.000	0.115	0.640
**DAG [36:2, (18:1, 18:1)]**	0.230	0.135	0.555	0.079
**LPC [18:2]**	0.229	0.000	0.381	0.390
**LPC [16:1]**	0.223	0.000	0.176	0.601
**SM [34:1]**	0.214	0.089	0.687	0.009
**PC [34:1, (18:1, 16:0)]**	0.207	0.216	0.483	0.094

**PC [32:1, (16:0, 16:1)]**	0.205	0.000	0.498	0.296
**TAG [52:5]**	0.203	0.087	0.597	0.113
**LPC [16:0]**	0.197	0.000	0.157	0.646
**CER [40:2]**	0.193	0.051	0.691	0.065
**TAG [56:5]**	0.190	0.100	0.627	0.083
**PC [33:1, (18:1, 15:0)]**	0.188	0.000	0.653	0.159
**PE-O [38:6, (18:2, 20:4)]**	0.187	0.000	0.130	0.684
**PC [36:4, (20:4, 16:0)]**	0.180	0.000	0.477	0.343
**PC-O [34:2, (16:1, 18:1)]**	0.175	0.074	0.563	0.188
**PE-O [36:4, (18:2, 18:2)]**	0.165	0.280	0.438	0.117
**TAG [54:3]**	0.157	0.205	0.635	0.003
**SE [43:2, (27:1, 16:1)]**	0.155	0.000	0.261	0.584
**TAG [54:4]**	0.149	0.122	0.622	0.107
**CER [40:1]**	0.147	0.239	0.544	0.071
**SM [40:2]**	0.138	0.000	0.313	0.549
**PC [36:5, (16:0, 20:5)]**	0.136	0.000	0.278	0.587
**PC-O [34:1, (17:0, 17:1)]**	0.133	0.000	0.191	0.676
**LPE [22:6]**	0.126	0.000	0.492	0.382
**PC [37:3, (20:3, 17:0)]**	0.122	0.000	0.296	0.583
**SM [42:2]**	0.113	0.237	0.536	0.114
**PC-O [34:2, (18:2, 16:0)]**	0.113	0.263	0.460	0.165
**PE-O [36:3, (18:1, 18:2)]**	0.111	0.470	0.263	0.155
**SM [32:1]**	0.110	0.000	0.342	0.548
**TAG [54:2]**	0.108	0.008	0.705	0.179
**PC-O [32:1, (16:1, 16:0)]**	0.095	0.000	0.369	0.535

**PC [38:5, (18:1, 20:4)]**	0.095	0.231	0.673	0.001
**SM [34:2]**	0.085	0.308	0.467	0.140
**PC-O [36:4, (16:0, 20:4)]**	0.084	0.222	0.661	0.033
**TAG [51:1]**	0.084	0.006	0.738	0.172
**PI [36:2, (18:1, 18:1)]**	0.077	0.000	0.311	0.612
**PC-O [36:3, (16:0, 20:3)]**	0.067	0.000	0.337	0.597
**PC [37:4, (17:0, 20:4)]**	0.066	0.417	0.472	0.045
**PC-O [34:2, (16:0, 18:2)]**	0.065	0.372	0.542	0.021
**PE-O [34:3, (16:1, 18:2)]**	0.063	0.506	0.282	0.149
**PC [36:2, (18:1, 18:1)]**	0.062	0.182	0.610	0.146
**SM [34:0]**	0.061	0.078	0.655	0.205
**PC [38:5, (22:5, 16:0)]**	0.057	0.105	0.745	0.094
**PC-O [32:0, (18:0, 14:0)]**	0.050	0.000	0.123	0.827
**LPE [18:2]**	0.035	0.000	0.399	0.566
**PC [38:6, (20:4, 18:2)]**	0.032	0.133	0.778	0.057
**SE [42:1, (27:1, 15:0)]**	0.031	0.377	0.519	0.072
**TAG [50:1]**	0.030	0.000	0.354	0.615
**TAG [49:1]**	0.027	0.080	0.681	0.212
**LPC [14:0]**	0.027	0.000	0.356	0.617
**PC [32:0, (16:0, 16:0)]**	0.025	0.348	0.510	0.117
**SE [45:4, (27:1, 18:3)]**	0.021	0.365	0.426	0.188
**CER [42:2]**	0.020	0.486	0.383	0.111
**PC [32:1, (18:1, 14:0)]**	0.014	0.000	0.378	0.608
**SE [45:3, (27:1, 18:2)]**	0.013	0.497	0.415	0.075
**PC [38:4, (22:4, 16:0)]**	0.010	0.000	0.413	0.576

**PI [34:1, (18:1, 16:0)]**	0.010	0.000	0.224	0.765
**PC-O [32:0, (17:0, 15:0)]**	0.009	0.000	0.232	0.759
**TAG [50:2]**	0.008	0.006	0.470	0.517
**PC [34:2, (18:1, 16:1)]**	0.007	0.379	0.565	0.049
**SE [45:2, (27:1, 18:1)]**	0.007	0.428	0.565	0.000
**TAG [48:3]**	0.006	0.218	0.649	0.126
**SE [45:1, (27:1, 18:0)]**	0.006	0.311	0.619	0.064
**SM [38:2]**	0.006	0.366	0.527	0.102
**ST [27:1]**	0.005	0.197	0.652	0.146
**PC [36:4, (18:2, 18:2)]**	0.005	0.274	0.590	0.131
**PC [34:2, (18:2, 16:0)]**	0.004	0.250	0.678	0.067
**PC-O [38:4, (18:1, 20:3)]**	0.004	0.084	0.660	0.253
**TAG [48:1]**	0.003	0.527	0.326	0.144
**TAG [52:3]**	0.002	0.292	0.617	0.088
**PC [30:0, (16:0, 14:0)]**	0.002	0.000	0.381	0.617
**PC-O [36:4, (18:2, 18:2)]**	0.001	0.001	0.535	0.464
**TAG [53:2]**	0.001	0.468	0.403	0.128
**LPC [22:6]**	0.001	0.544	0.363	0.093
**TAG [52:4]**	0.000	0.565	0.391	0.044
**PI [38:4, (18:0, 20:4)]**	0.000	0.500	0.418	0.082
**PC [36:2, (18:0, 18:2)]**	0.000	0.252	0.627	0.121
**TAG [50:3]**	0.000	0.504	0.489	0.007
**TAG [54:5]**	0.000	0.437	0.512	0.051
**SE [47:5, (27:1, 20:4)]**	0.000	0.369	0.564	0.067
**CER [38:1]**	0.000	0.370	0.431	0.199

**TAG [56:3]**	0.000	0.190	0.784	0.026
**SE [43:1, (27:1, 16:0)]**	0.000	0.348	0.607	0.045
**SE [44:1, (27:1, 17:0)]**	0.000	0.029	0.321	0.650
**TAG [48:2]**	0.000	0.517	0.340	0.143
**PC-O [38:4, (18:0, 20:4)]**	0.000	0.419	0.495	0.085
**TAG [56:4]**	0.000	0.504	0.415	0.081
**SE [49:7, (27:1, 22:6)]**	0.000	0.535	0.460	0.006
**PC-O [38:5, (18:1, 20:4)]**	0.000	0.450	0.430	0.120
**PC [34:3, (16:0, 18:3)]**	0.000	0.372	0.546	0.082
**TAG [50:4]**	0.000	0.399	0.521	0.079
**PC [38:4, (18:0, 20:4)]**	0.000	0.467	0.437	0.096
**SE [47:6, (27:1, 20:5)]**	0.000	0.453	0.454	0.093
**TAG [53:4]**	0.000	0.432	0.434	0.133
**PC [35:2, (17:0, 18:2)]**	0.000	0.310	0.496	0.195
**PC [36:3, (18:1, 18:2)]**	0.000	0.435	0.557	0.008
**DAG [36:3, (18:2, 18:1)]**	0.000	0.518	0.433	0.048
**SE [44:2, (27:1, 17:1)]**	0.000	0.374	0.548	0.078
**PC-O [36:3, (18:2, 18:1)]**	0.000	0.372	0.574	0.053
**TAG [51:2]**	0.000	0.554	0.353	0.093
**TAG [48:0]**	0.000	0.492	0.319	0.189
**TAG [54:7]**	0.000	0.260	0.648	0.092
**PC [32:2, (18:2, 14:0)]**	0.000	0.013	0.441	0.546
**PC [34:0, (18:0, 16:0)]**	0.000	0.507	0.443	0.050
**TAG [51:3]**	0.000	0.449	0.527	0.024
**PC-O [36:5, (16:1, 20:4)]**	0.000	0.352	0.485	0.163

**TAG [46:1]**	0.000	0.413	0.580	0.007
**CER [42:1]**	0.000	0.430	0.398	0.171
**PC [34:3, (18:2, 16:1)]**	0.000	0.502	0.471	0.027
**TAG [56:7]**	0.000	0.101	0.747	0.152
**SE [41:1, (27:1, 14:0)]**	0.000	0.393	0.426	0.181
**TAG [53:3]**	0.000	0.262	0.610	0.127

Supplementary Figure S1: Plasma concentration of the stable lipid classes after LF.

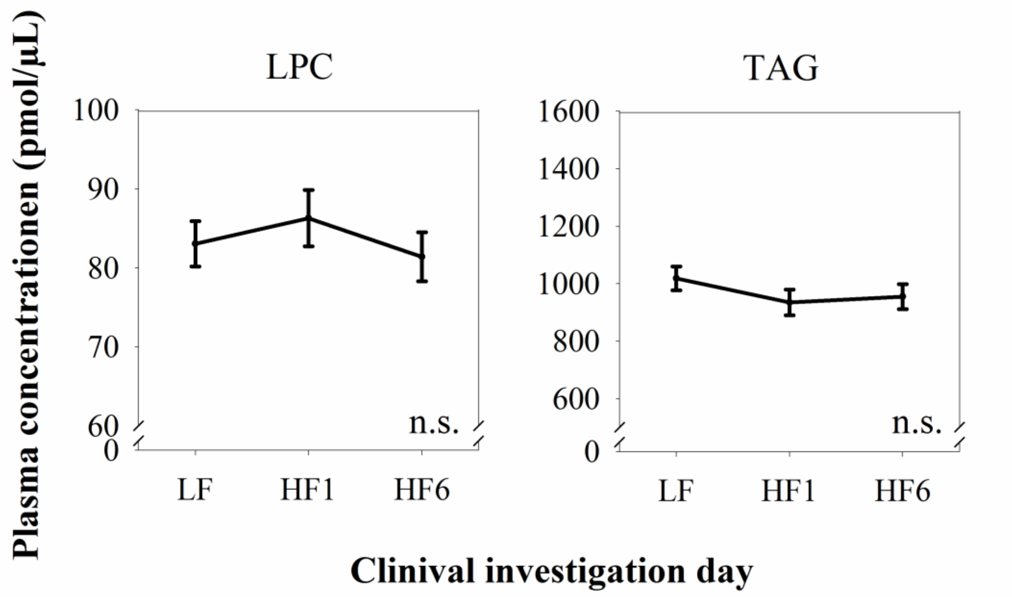

Supplementary Figure S2: Pie charts of class dependent species changes after normalization (LF, HF1, HF6).

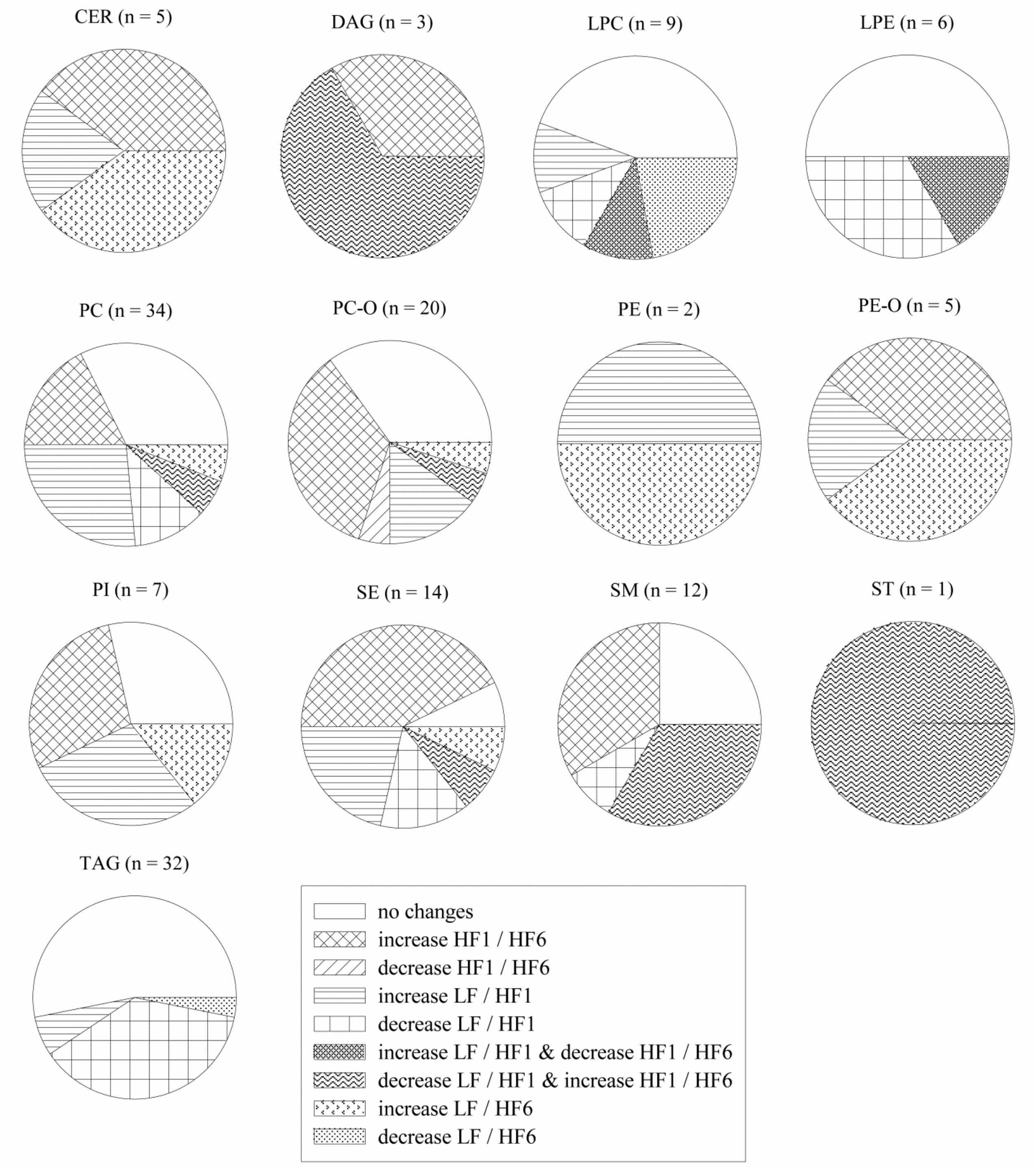

Supplementary Figure S3: Flexibility of lipid species (n=150).

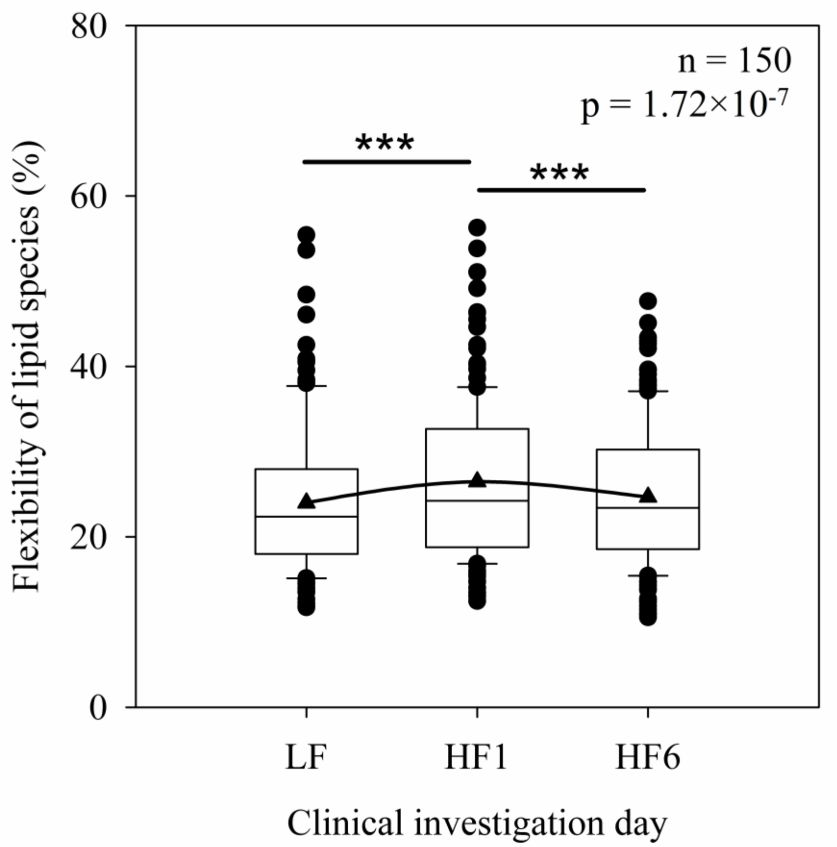

Supplementary Figure S4: Flow chart for the analysis of lipid species.

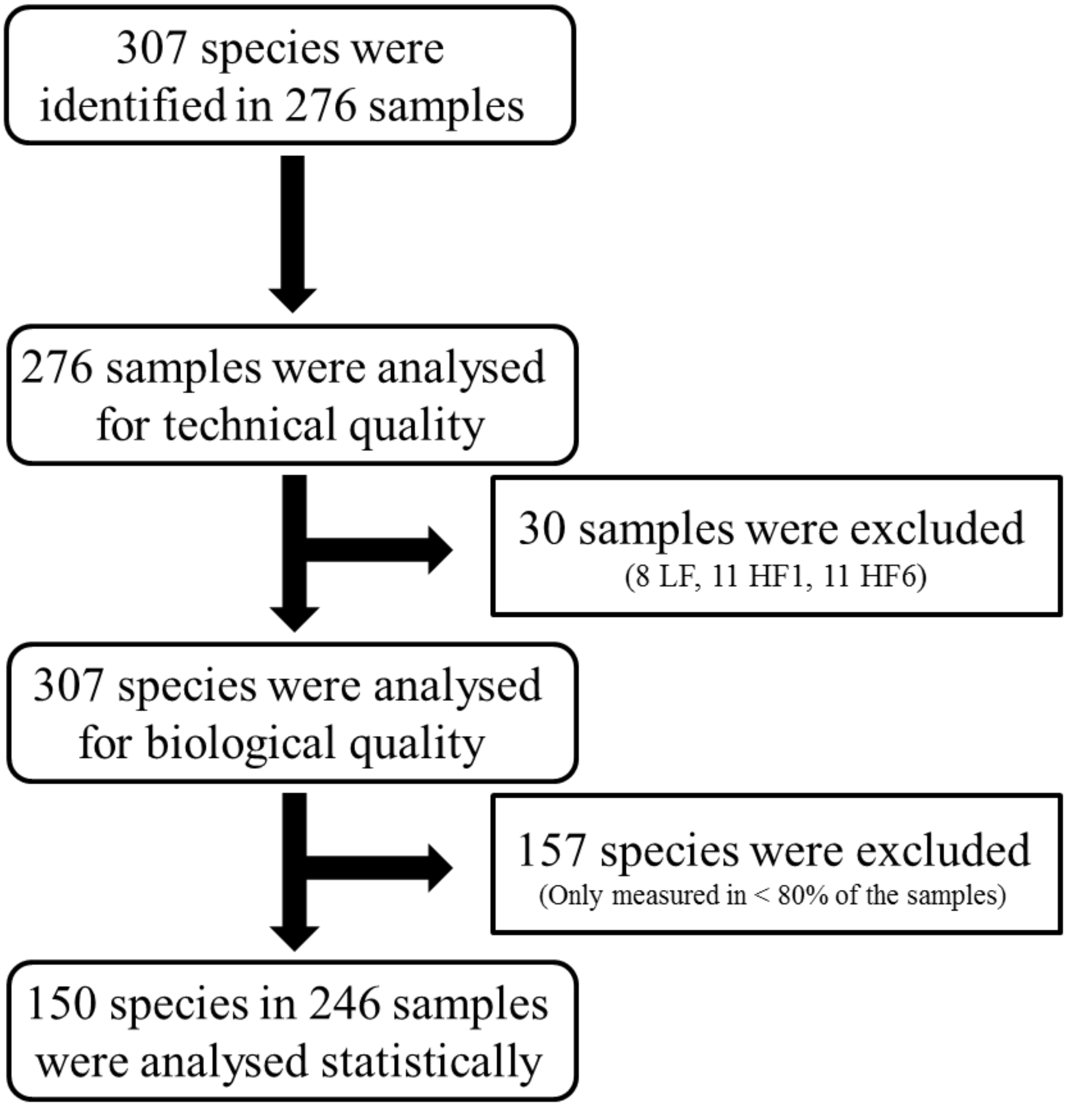

Supplement: Supplementary file 1 — Supplementary Material [file 41598_2017_3965_MOESM1_ESM.pdf]
